# Smartphone Ownership, Minors’ Well-being, and Parental Mediation Strategies. An Analysis in the Context of Social Media Influencers

**DOI:** 10.1007/s10964-024-02013-7

**Published:** 2024-05-23

**Authors:** Miguel Ángel Martín-Cárdaba, Mercedes Victoria Martínez Díaz, Patricia Lafuente Pérez, Javier García Castro

**Affiliations:** 1 Universidad Villanueva, Calle de la Costa Brava 2 y 6, 28034 Madrid, Spain; 2 National University of Distance Education (UNED), Calle Juan del Rosal, 10, 28040 Madrid, Spain

**Keywords:** Social media influencers, Parasocial relationship, Parental mediation strategies, Children and teenagers, Online risk

## Abstract

Although smartphone ownership among minors has become an important social phenomenon, its impact on children’s and adolescents’ well-being, as well as the mechanisms by which this might take place are not yet sufficiently well-established. To date, no research has examined the effect of smartphone ownership on the well-being of minors through the consumption of influencer-generated content, nor has it explored the effectiveness of the main prevention strategies employed by parents in this context. To fill those gaps, 800 Spanish minors (50% female) aged from 8 to 16 years old (*M* = 12.33, SD = 2.38) participated in a correlational study in which the ownership of electronic devices, the consumption of influencer generated content, the parasocial relationship with the influencer, and the most common parental mediation strategies were considered. The results showed a positive association between electronic device ownership and psychological discomfort, problematic usage, and imitation of dangerous behaviors. This association was mediated by the consumption of influencer-generated content and the parasocial relationship established by the minor with the influencer. Regarding preventive strategies, only active mediation was inversely related to poorer well-being indicators, however this positive effect significantly decreased when a smartphone or a similar electronic device was owned by the minor (vs. no owned). These findings contribute to the understanding of how smartphone ownership can affect the well-being of children, emphasizing the need for thoughtful consideration when deciding whether to provide smartphones to minors.

## Introduction

The increasing use of smartphones and similar electronic devices has been robustly linked to diminished well-being among children and adolescents (Busch & McCarthy, 2021). However, research investigating the impact of smartphone ownership on young individuals’ well-being remains scarce and has yielded conflicting findings, occasionally associating it with adverse outcomes (e.g. Dempsey et al., 2019) but producing inconclusive results in other instances (e.g., George et al., 2020). This lack of consistency in previous findings may stem from a greater emphasis on whether minors owned smartphones rather than on the types of content they consumed through these devices (Sun et al., 2023). Hence, further research is warranted to investigate the correlation between smartphone ownership and its impact, particularly emphasizing the consumption of specific content. One of the most popular types of content consumed by children through their electronic devices is content generated by influencers—public figures who foster strong, intimate connections with their followers, known as parasocial relationships—(Tolbert & Drogos, 2019). Even though the content published by influencers is not generally perceived by parents as particularly worrisome, especially when compared to more hazardous contents accessible on the Internet, such as explicit violence or pornography (Cornish, 2014), there are sufficient theoretical arguments supporting its potential negative influence on the younger population (Lowe-Calverley & Grieve, 2021; Panjrath & Tiwari, 2021). Thus, studying whether the consumption of influencer-generated content is associated with any detrimental consequences, the extent of these consequences, and the conditions under which they occur has become increasingly essential (Sharma et al., 2023). Furthermore, parents and educators frequently implement various protective measures, known as parental mediation strategies, when they are concerned about their children’s Internet usage (Valkenburg et al., 1999). However, while previous research has assessed the effectiveness of these strategies in broader Internet use contexts (Livingstone & Helsper, 2008), their efficacy in relation to device ownership, particularly within the context of influencer-generated content remains unexplored. Therefore, this study aims, firstly, to investigate the relationship between electronic device ownership, consumption of influencer-generated content, and potential adverse outcomes such as psychological distress, problematic usage patterns, or emulation of risky behaviors among children and adolescents. Secondly, it aims to examine the effectiveness of parental mediation strategies in mitigating these negative consequences when device ownership is transferred to minors (versus not), within the context of social media influencer consumption.

### Smartphone Use and Smartphone Ownership

The proportion of minors who are users and owners of smartphones is growing substantially (Rideout & Robb, 2019). According to data from the United States and Europe, early adolescents often get their first smartphone between the ages of 10 and 11 (Moreno et al., 2019). These data are cause for concern, as a significant body of previous research has consistently found a link between heavy smartphone use and lower well-being among children (Busch & McCarthy, 2021). Specifically, studies have shown an association between mobile phone use and a higher rate of depression (Boers et al., 2019), psychological distress (Twenge & Campbell, 2018), sleep disturbances (de Sá et al., 2023), academic underperformance (Lepp et al., 2014), and poorer quality in-person social interactions (George & Odgers, 2015). Even though much of the research has mainly focused on the implications of frequency of use and time spent on smartphones and similar devices, only a small number of studies have looked at the effects of youth smartphone ownership. However, the results have been mixed. Some studies have suggested negative effects stemming from smartphone ownership. For instance, a longitudinal study found children’s mobile phone ownership at age 9 associated with lower math and reading performances at age 13 (Dempsey et al., 2019). Additionally, smartphone ownership was linked to increased electronic media use in bed and later bedtimes in a cross-sectional study of teenagers aged 12 to 17, although it was not substantially correlated with sleep difficulties or depressive symptoms (Lemola et al., 2015). Furthermore, early smartphone ownership seems to have a detrimental effect on respondents’ self-assessed problematic conduct and intellectual capacity, but only for female respondents (Dempsey et al., 2020). Another study found that early smartphone access is negatively associated with adolescents’ math and language performance and creative digital media use. Similarly, owning a smartphone early in life appears to be linked with both greater smartphone pervasiveness in the subsequent years and greater risk of smartphone problematic use (Gerosa et al., 2024). However, other research indicated very little proof of ownership effects. A longitudinal study found that smartphone ownership among 9 to 15-year-olds does not appear to be contextually related with markers of well-being like academic progress or psychological discomfort 1–2 years later (George et al., 2020). Additionally, a recent study using a sample of low-income immigrant children who were overweight did not find statistically significant associations linking smartphone ownership, acquisition, and use, and markers of well-being, including academic performance, depressive symptoms, or sleep disturbances (Sun et al., 2023). Thus, the contradictory nature of these previous findings suggests that while focusing on ownership and usage time is relevant, it may be equally important to consider how children use smartphones and similar electronic devices, as well as the type of content they consume.

### Social Media Influencers

Engaging with social media is one of the most common uses of smartphones and electronic devices among children and adolescents (Jeong et al., 2016). Even though there are some alternative interpretations (e.g., Heffer et al., 2019), numerous studies suggest a negative relationship between social media use and adolescent psychological well-being (Barthorpe et al., 2020). It has been shown that social media use can adversely affect various aspects of adolescents’ lives, such as sleep quality (Woods & Scott, 2016), academic performance (Giunchiglia et al., 2018), family relationships, and social bonds (Allcott et al., 2020). However, the term “social media use” encompasses far too many different types of content and behaviors. Thus, given that different contents and uses of social networks (e.g., passive content consumption vs. communication and sharing emotions) have a different impact on children’s well-being (Liu et al., 2019), it is becoming necessary to further specify the type of activity to be studied. Within the context of social networks, influencers are one of the most relevant actors with whom teenagers can interact (Lajnef, 2023). Influencers can be defined as independent actors with the power to shape opinions and behaviors of their followers using social media platforms (Hudders et al., 2021). This ability to be influential lies in some of its characteristic attributes, such as attractiveness and credibility (Lou & Yuan, 2019). Another of the variables identified by previous research that allows explaining the particular influence capacity of these media personalities is the type of relationship that their followers establish with them, which has been named parasocial relationship.

The concept of *parasocial relationship* (Horton & Wohl, 1956) refers to the feeling of friendship and intimacy that a viewer develops toward a media character (i.e., celebrities, media figures or influencers) over the course of his or her continuous interaction. In the context of social networks, parasocial relationships are even more profound, since, unlike traditional mass media, social media makes it easier for users to communicate and interact with their favorite influencer (e.g., sharing, liking, commenting, replying, etc.) leading to more intimacy and improved reciprocity in the follower’s perception (Colliander & Dahlén, 2011; Yuan & Lou, 2020). In this way, the parasocial relationship that these people establish with their followers would facilitate the occurrence of influential processes, both at the cognitive, affective, and behavioral levels. For instance, a positive link between the perceived trustworthiness, expertise of the influencer and the parasocial relationship has been observed, showing that the greater the perceived intimacy with the influencer, the greater the trust the influencer can elicit (Lou & Kim, 2019). In this sense, the stronger the parasocial relationship that the follower experiences towards the influencer, the greater the probability that the former will be influenced by the latter (Hoffner & Bond, 2022). This unique trust and connection that followers experience towards influencers suggests that their content may have a significantly greater impact on their audiences than many other contents present on social media platforms (Su et al., 2021). For this reason, an increasing number of researchers are recognizing the need to pay special attention to the content published by influencers and the possible effect it could have on the well-being of their followers. For example, a recent study found experimentally that a group of women who were exposed to influencer images on Instagram experienced greater negative mood and body dissatisfaction compared to a control group exposed to neutral Instagram images (e.g., starfish in the sand) (Lowe-Calverley & Grieve, 2021). In the same way, another study has recently found that the adolescent girls and middle-aged women who followed fashion influencers were more dissatisfied with their body image and had lower self-esteem compared to those who did not (Panjrath & Tiwari, 2021). These results are consistent with more recent research showing how a high Instagram usage could lead to a decrease in psychological well-being (Garcia et al., 2022; Sherlock & Wagstaff, 2019; Staniewski & Awruk, 2022).

One of the most popular hypotheses that explain these findings argues that this *psychological discomfort* may be a consequence of followers comparing their bodies and lives with those exhibited by their favorite influencer (Hogue & Mills, 2019). This phenomenon, for which the term *facebook envy* was initially coined, refers to users that compare themselves with others causing a decrease in life satisfaction, especially among adolescents (Chou & Edge, 2012; Sagioglou & Greitemeyer, 2014; Tandoc et al., 2015). As previous research has shown, using social media to engage in harmful social comparisons was linked more strongly with depression than overall social media usage (Yoon et al., 2019). Moreover, a similar study revealed that Instagram use related to social comparisons with influencers was associated with lower levels of body appreciation (Pedalino & Camerini, 2022). Another reason why adolescents may see their emotional well-being diminished as a consequence of their relationship with the influencer could be related to a phenomenon called *fear of missing out* (FOMO), which is both a constant apprehension that one could be missing out on wonderful experiences that others are having and a desire to always be connected with what they are doing or sharing (Milyavskaya et al., 2018). This fear seems to be related to more anxiety states, negative emotions, discomfort and even the abandonment of important duties and healthy habits (Oberst et al., 2017). Therefore, it could be expected that the stronger the connection with the influencer, the greater the frustration experienced when followers cannot attain the physical appearance or lifestyle of their role models, as well as when they cannot keep up with all the content they publish (Hoffner & Bond, 2022).

Similarly, the negative effect on the well-being of minors could be related to a *problematic use* of content generated by influencers. For example, the *displacement hypothesis* (Kraut et al., 1998) proposes that excessive social media consumption could lead to an emotional well-being impairment because the time that may be spent on in-person social interactions, protective, health-promoting habits like physical activity or educational and extracurricular activities is instead spent doing this sedentary activity. Thus, a high propensity to consume content generated by their favorite influencers could result in children having much less time and motivation to do many of these other desirable and beneficial activities.

Finally, another reason why influencer-generated content could pose a risk to the well-being of their younger followers is that they might want to imitate some *dangerous behaviors* exhibited by their role models. Some of these risky behaviors are related to the social media challenges. While some challenges possess positive underlying intentions and are relatively safe, many others involve health risk behaviors (e.g., the Cinnamon Challenge: ingesting a tablespoon of cinnamon without liquids; the Tide Pod Challenge: ingesting a Tide Pod, containing chemicals; and the Kiki Challenge: dancing beside a moving vehicle). These challenges, which gain visibility when undertaken by influencers and are subsequently imitated by many of their followers, have resulted in serious health consequences including alcohol abuse (Hendriks et al., 2020), unsafe sexual behaviors (Yusuf, 2021), aspiration, poisoning, motor vehicle accidents, and even death (Ward et al., 2021). In this regard, *Social Learning Theory* (Bandura, 1977) predicts that children are more likely to imitate a character’s behavior the more they like that character, and also S*ocial Network Contagion Theory* (Scherer & Cho, 2003) explains that minors are more likely to reproduce the behavior of a source who they could identify themselves with better (such as influencer), even if that behavior is undesirable or harmful. Additionally, the *Facebook Influence Model* (Moreno & Whitehill, 2014) suggests peer influence mechanisms that could make teenagers more likely to engage in dangerous behaviors as a result of using social media.

All these potential negative effects are even more concerning considering the vulnerability of the target population. Children and teenagers at this age have not fully developed their cognitive abilities, rendering them particularly susceptible to the potential virtual risks (Haddon et al., 2020). It has been shown that children and adolescents are more susceptible to persuasion in social media (Van Reijmersdal & Van Dam, 2020) and they will be more affected by digital media than adults (Tesar & Hood, 2019). Specifically, previous research has shown that the relationship between social media use estimates and life satisfaction ratings is more negative in younger adolescents than in adults. Furthermore, longitudinal analyses of 17,409 participants (10–21 years old) suggested specific developmental periods of sensitivity to social media during adolescence (Orben et al., 2022).

### Parental Mediation Strategies

The specific vulnerability of children and adolescents as well as the presence of risks on the Internet in general and on Social Network Sites in particular have led parents to seek strategies to protect their children from such threats (Geržičáková et al., 2023). Within this context, parental mediation refers to a set of strategies used to mitigate children’s negative uses of the media and its negative consequences (Sasson & Mesch, 2019). These different strategies can be interpreted as a reflection of different ideologies and beliefs about children´s media use (Modecki et al., 2022). Current classifications of mediation strategies related to the use of the Internet (Livingstone et al., 2017) are an evolution of the first classifications that were made in the context of traditional media (Valkenburg et al., 1999). Initially, these strategies could be summarized into three main categories: active mediation, restrictive mediation, and co-viewing. *Active (or instructive) mediation* includes discussing, evaluating, and interpreting the media content with the child. *Restrictive* strategies consist in setting rules, timetables, and regulations about what, when and how much media content can be accessed. Thus, restrictive mediation could be split into *access restriction* (how much and when a particular media can be used) and *content restriction* (which content can be seen). Finally, *co-viewing* implies that parents use the media together with the child, although parents might not necessarily discuss the content (Nikken & Jansz, 2014). Recently, expanded classifications for mediation strategies have been suggested, including *technical mediation* (installation of software to control, filter or block certain online content) and *monitoring* (checking on the child’s online activities after use) (Livingstone et al., 2011; Mitchell et al., 2005; Symons et al., 2017a).

Due to a growing interest among parents, educators and researchers about the effectiveness of each of these strategies in reducing the risks associated with children’s Internet use, an increasing amount of research is being conducted (Chen & Shi, 2019; Meeus et al., 2018; Young & Tully, 2022). With respect to active mediation, previous research has shown that encouraging, sharing, or discussing the child’s online activities is effective in decreasing the length of Internet use (Sasson & Mesch, 2014) and the likelihood of being engaged in online risks (Young & Tully, 2022), such as contact risks (Shin & Ismail, 2014), privacy disclosure (Kang et al., 2022; Lwin et al., 2008), and cyberbullying (Chang et al., 2015). In addition, active parental mediation tends to prevent children’s exposure to online risks without reducing their positive online opportunities (Duerager & Livingstone, 2012). Regarding restrictive strategies, some studies have suggested that restrictive mediation can be an effective way to protect children from harmful media influences (Lee, 2012; Lwin et al., 2008; Marcum et al., 2010). For example, parental restrictions could prevent children’s exposure to online risks, because it reduces general Internet activity (Kalmus et al., 2015). However, other research has found that there are fewer promising results and even results that go in the opposite direction. For example, it has been shown that restrictive mediation strategies could be associated with a greater number of risky behaviors (Sasson & Mesch, 2014). Similarly, adolescents who reported more cybervictimization were among those who were more likely to report higher levels of parental restrictions (Baldry et al., 2019; Wright, 2016). Moreover, although these restrictive strategies can be effective reducing the time children spend on media, they may also foster parent-child conflicts (Beyens & Beullens, 2017), hinder the acquisition of digital skills (López-de-Ayala et al., 2021) and could even increase the likelihood of children being addicted to media (Chen & Shi, 2019). Regarding co-viewing, monitoring and technical mediation, the evidence is also conflicting. Some studies have shown that co-using may reduce children’s exposure to online content risks (Kirwil, 2009; Wright, 2016) and has been associated with enhanced feelings of closeness between parents and children (Courtois & Nelissen, 2018). In a similar vein, monitoring the websites visited by youngsters and installing monitoring software reduce the likelihood of online victimization (Mesch, 2016; Navarro et al., 2013). However, other researchers have found that methods such as co-usage, technological restrictions, and monitoring are ineffective in protecting children from Internet risks (Livingstone & Helsper, 2008).

The conflicting nature of these previous findings suggests that more research is needed to better understand when, in what circumstances and for whom each strategy is most effective. One of the possible reasons for this disparity in results is that the concept of *social media use* is too broad and includes a wide range of different content and behaviors (e.g., self-presentation management, information-sharing, emotion-sharing, passive content usage). For this reason, this research aims to further explore the effectiveness of parental mediation strategies with respect to a specific type of content and activity that has not been studied to date. Also, providing a smartphone or similar device for a minor’s personal use is an important parental decision that can have relevant consequences for children regarding their use and access to the Internet and social media. On the one hand, the possession of smartphones by children could increase the time and intensity of Internet and social network use due to greater accessibility. On the other hand, it could affect the effectiveness of parental mediation strategies, making parental control more difficult (Hwang et al., 2017; Nikken & Jansz, 2014). However, previous research has not studied the role that the ownership of an electronic device by children and adolescents might play on other variables such as the time spent consuming influencer-generated content, or its moderator role between the parental mediation strategies and possible negative consequences.

## The Current Study

Although previous literature has investigated the negative effects of smartphone use on children’s and adolescents’ well-being, findings regarding smartphone ownership are still scarce and somewhat conflicting. To date, no research has examined the impact of smartphone ownership on the well-being of minors through the consumption of influencer-generated content, nor has it explored the effectiveness of the main prevention strategies employed by parents in this context. Therefore, the aim of the present study was twofold: (1) to investigate the possible connection between minors’ ownership of electronic devices, the consumption of influencer-generated content, the parasocial relationship between minors and influencers, and adverse consequences for well-being of minors, and (2) to explore whether commonly employed preventive strategies by parents could effectively mitigate these negative consequences among children and adolescents as well as evaluate the role that electronic devices ownership might play within this context. Furthermore, the age of minors was considered in both approaches. It was hypothesized that the ownership of electronic devices (e.g., smartphones) would be associated with poorer well-being indicators (psychological discomfort, problematic usage, and imitation of dangerous behavior) due to an increase in consumption of influencer-generated content and the parasocial relationship with the influencer. It was anticipated that the results would be moderated by age, with younger participants expected to report poorer well-being indicators. In addition, the examination of the moderating role of electronic device ownership on parental mediation strategies effectiveness was mainly exploratory since, to our knowledge, no previous study has addressed this research question yet; therefore, no specific hypothesis was formulated.

## Methods

### Participants and Procedure

A total of 800 minors, aged from 8 to 16 years old (*M*_age_ = 12.33 years, SD = 2.38; 50% female), participated voluntarily in exchange for a financial reward in an online self-administered questionnaire (CAWI system). The participants were recruited in large and small areas in Spain, in which 17 autonomous communities were well represented (e.g., Andalucia, 16.8%, Madrid, 19.9%, or Catalonia, 15.1%). The study was approved by the Ethical Committee of the lead author’s university of affiliation. All minors had parental consent to participate in the survey. A sensitivity analysis using G*Power (Erdfelder et al., 1996) indicated that a sample of 800 participants could detect an effect of *f*^2^ = 0.01 with 80% power in a linear multiple regression, fixed model, and *R*^2^ increase. No missing data were found.

The survey was divided into two parts. Initially, parents read the information sheet, informed consent, and completed the demographic items; then they instructed their children to respond to the survey autonomously and independently. However, minors could be assisted by them if they had any questions. Secondly, the children were asked to identify their favorite influencer and indicate the social media platform used to follow them (e.g., Instagram, Snapchat, Tik Tok, Facebook) before proceeding to the scales. Finally, participants were debriefed, thanked, and dismissed.

### Measures

#### Consumption of influencer-generated content

In order to understand the amount of time that minors spent watching influencers, they were asked to report the time they considered spent following influencers on weekends, and on school days by using a 2-item scale (Tolbert & Drogos, 2019). Response options included 1 (no time), 2 (0–30 min), 3 (31–60 min), 4 (61 min–2 h), 5 (more than 2 h), 6 (more than 3 h), 7 (more than 4 h). The responses were averaged (*M* = 3.35, SD = 1.16). Higher scores indicated higher consumption.

#### Parasocial relationship

A 6-item scale (adapted from Eyal & Cohen, 2006) was used to measure the intensity of parasocial relationship with their preferred influencers (*α* = 0.89). The participants were asked to identify their favorite influencer and to think of him or her when answering the items on the scale. Sample items from the scale includes: “my favorite influencer is like a friend to me,” “my favorite influencer is like an advisor to me,” “my favorite influencer is like a member of my family.” The participants responded using a 7-point Likert scale ranging from 1 (strongly disagree) to 7 (strongly agree). The responses were averaged (*M* = 5.02, SD = 1.17). Higher scores indicated a stronger parasocial relationship with the influencer (for a detailed description of all measures see Supplementary Materials; Martínez, 2024).

#### Parental mediation strategies

To assess parental mediation strategies, items from previous research were used (e.g., Geržičáková et al., 2023; Livingstone et al., 2011; Padilla-Walker et al., 2012; Valkenburg et al., 2013). On the one hand, minors expressed their level of agreement with different statements using a 7-point Likert scale ranging from 1 (strongly disagree) to 7 (strongly agree). To assess *active* mediation, the item “my father/mother is concerned and explains to me the dangers of social media” (*M* = 5.95, SD = 1.15) was used; for *co-viewing*, “my father/mother sits with me to see my favorite influencers” (*M* = 5.09, SD = 1.57), for *restrictive time* mediation, “my father/mother limits the time I spend viewing content on social networks” (*M* = 4.14, SD = 2.11), and for *restrictive content* mediation, “my father/mother has forbidden me to see a specific influencer” (*M* = 5.22, SD = 1.74). On the other hand, parents were asked to answer using a dichotomous option (1 no, 2 yes) if the device used by minors has some control software installed (i.e., *technical mediation*, Symons et al., 2017b), and/or if they reviewed the content that their children viewed on social networks (i.e., *monitoring*, Chang et al., 2015). Specifically, parents completed the next items: “does the electronic device regularly used by the child have any kind of parental control software (e.g., Qustodio, Family Link) to monitor his or her activity?” and “do you usually take the time to review the content your child views on social networks?” The responses showed that almost half of the participants (49.7%) had software to control the use of device, and 69.6% of parents reported checking the content viewed by their children (2.6% and 1.4% did not answer the questions respectively).

#### Ownership of electronic device

A single item was used to measure the ownership of electronic devices. The parents were asked to complete the following statement: “is the device on which your child regularly views influencers and social media content owned by your child or for your child’s exclusive use?” the participants responded using a dichotomous option (1 no owned, 2 owned). The responses showed that 77.5% of children owned an electronic device exclusively for their use.

#### Psychological discomfort

A 4-item scale was used to measure the intensity of psychological discomfort (*α* = 0.89; adapted from Verduyn et al., 2015). Sample items included: “after watching my favorite influencer, I feel a little worse about my body,” “after watching my favorite influencer, I feel a little worse about myself,” or “after watching my favorite influencer, I feel a little worse about my life.” the participants responded using a 7-point Likert scale ranging from 1 (strongly disagree) to 7 (strongly agree). The responses were averaged (*M* = 3.51, SD = 1.73). Higher scores indicated higher psychological discomfort.

#### Problematic usage

To estimate the degree to which minors perceived that they engage in excessive or compulsive consumption of influencer-generated content that prevented them from performing other tasks considered to be beneficial such as sleeping, doing homework, and spending time with friends, a 4-item scale was used (*α* = 0.86; adapted from Tutgun-Ünal & Deniz, 2015). Items included, “I feel that I spend more time watching/following my favorite influencer than I should,” “sometimes I am watching my favorite influencer when I should be sleeping,” “sometimes I am watching my favorite influencer when I should be doing my homework,” and “I have sometimes stopped playing with my friends so that I could have more time to follow my favorite influencer.” The participants responded using a 7-point Likert scale ranging from 1 (strongly disagree) to 7 (strongly agree). Responses were averaged (*M* = 4.07, SD = 1.60). Higher scores indicated more problematic usage.

#### Dangerous behaviors

A single item was used to measure the actual occurrence of dangerous behaviors. The participants were asked to complete the following item: “I have sometimes imitated the dangerous or inappropriate behavior of my favorite influencer.” The participants responded using a 7-point Likert scale ranging from 1 (strongly disagree) to 7 (strongly agree). The responses were averaged (*M* = 3.43, SD = 2.06). Higher scores indicated the presence of more dangerous behaviors.

## Results

### Descriptive Analysis

As Table 1 shows, bivariate correlations were calculated to explore the relationship between variables. However, because correlations do not control for confounding influences, regression analysis were used to test the hypotheses.

**Table 1:** Bivariate correlations between all variables

	*M*	SD	1	2	3	4	5	6	7	8	9	10	11	12	13	14
1. Age	12.33	2.38	1	0.001	0.181**	0.012	−0.028	−0.141**	−0.250**	−0.224**	−0.134**	−0.214**	0.378**	−0.018	0.074*	−0.002
2. Sex	1.50	0.50		1	−0.066	−0.004	0.021	0.018	−0.014	−0.085*	0.009	0.028	0.024	−0.015	−0.031	−0.123**
3. Consumption IGC	3.35	1.15			1	0.245**	0.033	0.012	−0.133**	0.003	−0.086*	−0.073*	0.233**	0.210**	0.358**	0.145**
4. Parasocial relationship	5.15	1.11				1	0.135**	0.238**	0.117**	0.183**	0.162**	0.154**	0.108**	0.475**	0.463***	0.378**
5. Active mediation	5.95	1.16					1	0.374**	0.321**	0.153**	0.109**	0.116**	0.006	−0.163**	−0.118**	−0.168**
6. Coview mediation	5.09	1.57						1	0.321**	0.334**	0.215**	0.250**	−0.075*	0.079*	0.049	0.073*
7. Restrictive time mediat.	5.22	1.74							1	0.445**	0.311**	0.313**	−0.148**	0.086*	0.017	0.105**
8. Restrictive content mediat.	4.14	2.11								1	0.296**	0.233**	−0.110**	0.327**	0.277**	0.354**
9. Technical mediation	1.49	0.50									1	0.391**	−0.016	0.151**	0.083*	0.153**
10. Monitoring	1.69	0.46										1	−0.067	0.109**	0.037	0.091*
11. Ownership of device	0.77	0.41											1	0.065	0.140**	0.064
12. Psychological discomfort	3.51	1.73												1	0.789**	0.752**
13. Problematic usage	4.07	1.61													1	0.683**
14. Dangerous behavior	3.43	2.07														1

*p < 0.05; **p < 0.01

### Mediation Analysis

**Fig. 1:**
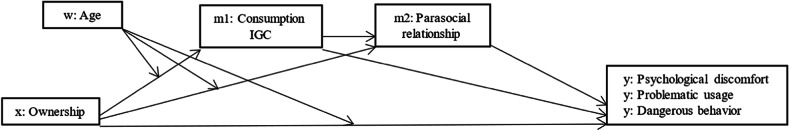
Indirect effect of ownership and age on psychological discomfort, problematic usage, and imitation of dangerous behaviors through consumption of influencer-generated content and parasocial relationship

To test whether the ownership of an electronic device (e.g., smartphones) is associated with poorer well-being indicators due to an increase in consumption of influencer-generated content and the parasocial relationship with the influencer, and whether this path is moderated by age, a double mediation moderated model was run by using the model 85 of SPSS PROCESS Macro provided by Hayes (2018) along with bootstrapping test (*n* boots = 5000). As can be seen in Fig. 1, the ownership of the device (1 not owned, 2 owned) was included as predictor, the age was included as moderator (centered), consumption of influencer-generated content (centered) and parasocial relationship (centered) were the mediator variables, and psychological discomfort, problematic usage, and dangerous behaviors were the outcome measures respectively.

As Table 2 shows, the analysis revealed significant indirect effects of ownership on psychological discomfort, problematic usage, and dangerous behavior, via influencer-generated content and parasocial relationship regardless of age. So, the first hypothesis was partially supported.

**Table 2:** Effects of ownership and age on the dependent variables through the consumption of influencer generated-content (IGC) and the parasocial relationship (PSR)

	*B*	SE	*t*	*p*	95% CIs
Consumption of influencer-content					
Ownership	**0.53**	**0.11**	**4.58**	**0.001**	**0.30, 0.76**
Age	**0.05**	**0.01**	**2.91**	**0.004**	**0.01, 0.08**
Interaction (Ownership × Age)	0.01	0.04	0.07	0.937	−0.08, 0.09
Parasocial relationship					
Ownership	0.19	0.12	1.59	0.112	−0.04, 0.42
IGC	**0.23**	**0.03**	**6.41**	**0.001**	**0.16, 0.30**
Age	−0.02	0.01	−1.56	0.119	−0.06, 0.01
Interaction (IGC × Age)	−0.04	0.05	−0.81	0.416	−0.13, 0.05
Psychological discomfort					
Ownership	−0.12	0.15	−0.79	0.424	−0.43, 0.18
IGC	**0.15**	**0.04**	**3.25**	**0.001**	**0.06, 0.25**
PSR	**0.70**	**0.04**	**15.28**	**0.001**	**0.61, 0.79**
Age	−0.03	0.02	−1.21	0.227	−0.07, 0.02
Interaction (Ownership × Age)	−0.11	0.06	−1.79	0.073	−0.22, 0.01
Index of moderated mediation (Owner-IGC)	0.01	0.01			−0.01, 0.01
Index of moderated mediation (Owner-PSR)	0.02	0.03			−0.09, 0.03
Index of moderated mediation (Owner-IGC-PSR)	0.01	0.01			−0.01, 0.02
Younger (−1 SD)	**0.09**	**0.02**			**0.04, 0.13**
Older (+1 SD)	**0.09**	**0.03**			**0.02, 0.17**
Problematic usage					
Ownership	0.01	0.14	0.04	0.961	−0.27, 0.28
IGC	**0.35**	**0.04**	**7.93**	**0.001**	**0.26, 0.46**
PSR	**0.57**	**0.04**	**13.58**	**0.001**	**0.49, 0.65**
Age	0.01	0.02	0.48	0.624	−0.03, 0.05
Interaction (Ownership × Age)	−0.08	0.05	−1.53	0.124	−0.19, 0.02
Index of moderated mediation (Owner-IGC)	0.01	0.02			−0.03, 0.03
Index of moderated mediation (Owner-PSR)	−0.02	0.02			−0.08, 0.03
Index of moderated mediation (Owner-IGC-PSR)	0.01	0.01			−0.01, 0.01
Younger (−1 SD)	**0.06**	**0.01**			**0.04, 0.11**
Older (+1 SD)	**0.07**	**0.03**			**0.02, 0.14**
Dangerous behaviors					
Ownership	−0.01	0.20	−0.05	0.960	−0.40, 0.38
IGC	0.09	0.06	1.53	0.126	−0.03, 0.21
PSR	**0.69**	**0.05**	**11.71**	**0.001**	**0.57, 0.81**
Age	−0.02	0.03	−0.57	0.568	−0.07, 0.04
Interaction (Ownership × Age)	−0.06	0.07	−0.88	0.374	−0.21, 0.08
Index of moderated mediation (Owner-IGC)	0.01	0.01			−0.01, 0.01
Index of moderated mediation (Owner-PSR)	0.03	0.03			−0.08, 0.03
Index of moderated mediation (Owner-IGC-PSR)	0.01	0.01			−0.01, 0.01
Younger (−1 SD)	**0.08**	**0.02**			**0.04, 0.13**
Older (+1 SD)	**0.08**	**0.03**			**0.01, 0.16**

Data in bold indicate statistically significant results

### Moderation Analysis

To test whether the ownership of the electronic device [e.g., smartphone] moderates the relationship between the parental mediation strategies and the negative consequences, two statistical analyses were run.

Firstly, to test which parental mediation strategies would be statistically associated with negative consequences, a hierarchical linear regression was conducted. Sex, age, parental mediation strategies (i.e., active, coviewing, restrictive-time, restrictive-content, technical mediation and monitoring), and electronic device ownership were incorporated as predictors. As Table 3 shows, results revealed a significant and negative relationship between the active mediation, and psychological discomfort, *β* = −0.24, *p* < 0.001, problematic usage, *β* = −0.17, *p* < 0.001, and dangerous behaviors, *β* = −0.25, *p* < 0.001. This indicates that minors who reported that their parents spent time talking to them about the dangers and threats of the Internet, also reported less psychological discomfort, problematic usage, and dangerous behaviors. Additionally, results revealed a significant and positive relationship between the restrictive content mediation, and psychological discomfort, *β* = 0.35, *p* < 0.001, problematic usage, *β* = 0.35, *p* < 0.001, and dangerous behaviors, *β* = 0.37, *p* < 0.001. This indicates that minors who reported that their parents forbidden them to see a specific influencer, also reported more psychological discomfort, problematic usage, and dangerous behaviors. Finally, a significant and positive relationship was found between the ownership of an electronic device and psychological discomfort, *β* = 0.09, *p* < 0.01, problematic usage, *β* = 0.14, *p* < 0.001, and dangerous behaviors, *β* = 0.09, *p* < 0.01. This indicates that minors who own their device also reported experiencing more negative consequences compared to those who did not own one.

**Table 3:** Hierarchical regression analysis on psychological discomfort, problematic usage, and dangerous behaviors

	Psychological discomfort				Problematic usage				Dangerous behaviors			
	*B*	SE	95% BCI	*β*	*B*	SE	95% BCI	*β*	*B*	SE	95% BCI	*β*
Age	0.03	0.02	−0.02, 0.08	0.04	**0.06**	**0.03**	**0.01, 0.11**	0.09*	0.06	0.03	−0.01, 0.11	0.06
Sex	0.05	0.11	−0.17, 0.27	0.01	−0.02	0.11	−0.22, 0.20	−0.01	−**0.38**	**0.01**	**−0.64**, **−0.12**	−0.09*
PMS												
Active	**−0.36**	**0.05**	**−0.46**, **−0.26**	−0.24***	**−0.24**	**0.05**	**−0.33**, **−0.13**	−0.17***	**−0.45**	**0.06**	**−0.58**, **−0.33**	−0.25***
Co-viewing	0.05	0.04	−0.03, 0.13	0.05	0.03	0.04	−0.04, 0.11	0.03	0.05	0.05	−0.04, 0.15	0.04
Restrictive time	−0.01	0.04	−0.09, 0.07	−0.01	−0.06	0.04	−0.13, 0.02	−0.06	0.01	0.05	−0.08, 0.10	0.01
Restrict. content	**0.29**	**0.03**	**0.23, 0.35**	0.35***	**0.26**	**0.03**	**0.21, 0.32**	0.35***	**0.36**	**0.04**	**0.29, 0.43**	0.37***
Technical	−0.12	0.11	−0.34, 0.10	−0.04	−0.07	0.11	−0.28, 0.14	−0.02	−0.18	0.12	0.45, 0.07	−0.05
Monitoring	−0.18	0.12	−0.42, 0.07	−0.05	0.01	0.12	−0.28, 0.23	0.01	−0.13	0.15	−0.42, 0.14	−0.03
Ownership	**0.42**	**0.15**	**0.42, 0.14**	0.10**	**0.54**	**0.14**	**0.26, 0.82**	0.14***	**0.46**	**0.17**	**0.12, 0.80**	0.09**
*R*^2^	0.170				0.145				0.203			
*F* (9, 790)	17.23***				14.28***				21.39***			

Data in bold indicate statistically significant results

*p < 0.05; **p < 0.01; ***p < 0.001

Secondly, as active mediation was the sole approach that exhibited positive effects on the dependent variables, it was evaluated whether ownership of an electronic device moderates the relationship that active mediation displays towards negative consequences, and whether such a relationship was moderated by age. For that purpose, a linear regression analysis including active mediation (centered), ownership of device (1 not owned, 2 owned), age (centered), and the interaction between them on each of the negative consequences was run. A moderation bootstrapping test (*n* boots = 5000) using Model 3 of SPSS PROCESS Macro provided by Hayes (2018) was selected. As Table 4 shows, the moderation analysis revealed a significant main effect of active mediation on psychological discomfort, problematic usage, and dangerous behavior, indicating fewer negative consequences for those participants who reported a high active mediation. The ownership showed a significant main effect on problematic usage, with minors who owned electronic devices reporting more issues, such effect were not statistically significant for psychological discomfort and dangerous behaviors. Finally, the main effect of age was marginally significant on problematic usage, indicating more problems for older (vs. younger) children, however no significant effects were found on psychological discomfort or dangerous behavior.

**Table 4:** Effects of active mediation, age and ownership of mobile on the dependent variables

	*B*	SE	*t*	*p*	95% CIs
Psychological discomfort					
Active	−**0.96**	**0.32**	−**2.94**	**0.003**	−**1.60**, −**0.32**
Ownership	0.20	0.17	1.13	0.255	−0.14, 0.54
Interaction (Active × Ownership)	**0.39**	**0.17**	**2.29**	**0.022**	**0.05, 0.73**
No owned	−**0.50**	**0.12**	−**4.11**	**0.001**	−**0.74**, −**0.26**
Owned	−**0.18**	**0.05**	−**3.27**	**0.001**	−**0.30**, −**0.07**
Age	0.19	0.12	1.52	0.127	−0.05, 0.44
Interaction (Active × Age)	−0.05	0.12	−0.42	0.674	−0.29, 0.19
Interaction (Owner × Age)	−0.13	0.07	−1.90	0.057	−0.26, 0.01
Younger (−1 SD)	**0.49**	**0.17**	**2.82**	**0.005**	**0.15, 0.84**
Older (+1 SD)	−0.13	0.29	−0.45	0.647	−0.71, 0.44
Interaction (Active × Owner × Age)	0.01	0.06	0.20	0.841	−0.11, 0.14
Problematic usage					
Active	−**0.70**	**0.30**	−**2.29**	**0.022**	−**1.30**, −**0.10**
Ownership	**0.39**	**0.16**	**2.35**	**0.019**	**0.06, 0.71**
Interaction (Active × Ownership)	0.29	0.15	1.82	0.067	−0.02, 0.60
No owned	−**0.37**	**0.11**	−**3.29**	**0.001**	−**0.60**, −**0.15**
Owned	−**0.11**	**0.05**	−**2.22**	**0.026**	−**0.22**, −**0.01**
Age	**0.19**	**0.11**	**1.66**	**0.097**	−**0.04, 0.43**
Interaction (Active × Age)	−0.03	0.11	−0.26	0.787	−0.25, 0.19
Interaction (Owner × Age)	−0.10	0.06	−1.58	0.113	−0.22, 0.02
Interaction (Active × Owner × Age)	0.01	0.06	0.18	0.854	−0.11, 0.13
Dangerous behaviors					
Active	−**1.14**	**0.39**	−**2.93**	**0.003**	−**1.91**, −**0.38**
Ownership	0.28	0.21	1.35	0.175	−0.13, 0.71
Interaction (Active × Ownership)	**0.45**	**0.20**	**2.21**	**0.027**	**0.05, 0.85**
No owned	−**0.55**	**0.14**	−**3.79**	**0.001**	−**0.84**, −**0.27**
Owned	−**0.24**	**0.06**	−**3.57**	**0.001**	−**0.37, 0.11**
Age	0.12	0.15	0.82	0.409	−0.17, 0.42
Interaction (Active × Age)	−0.14	0.14	−1.00	0.316	−0.44, 0.14
Interaction (Owner × Age)	−0.08	0.08	−1.07	0.286	−0.24, 0.07
Interaction (Active × Owner × Age)	0.06	0.07	0.86	0.389	−0.08, 0.22

Data in bold indicate statistically significant results

**Fig. 2:**
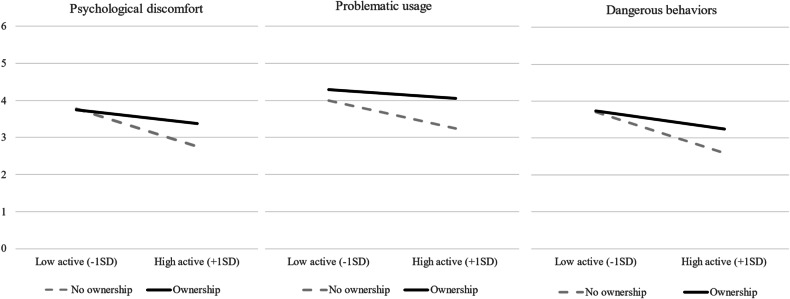
Psychological discomfort, problematic usage, and imitation of dangerous behaviors as a function of active mediation and ownership

Interestingly, a two-way interaction between active mediation and device ownership were found on psychological discomfort, dangerous behavior, and marginally significant on problematic usage (see Fig. 2). The positive effect of the active mediation on negative consequences for those minors who did not own the device was stronger compared to those who did own one, across all dependent variables. Finally, a two-way interaction between ownership and age was found on psychological discomfort.

**Fig. 3:**
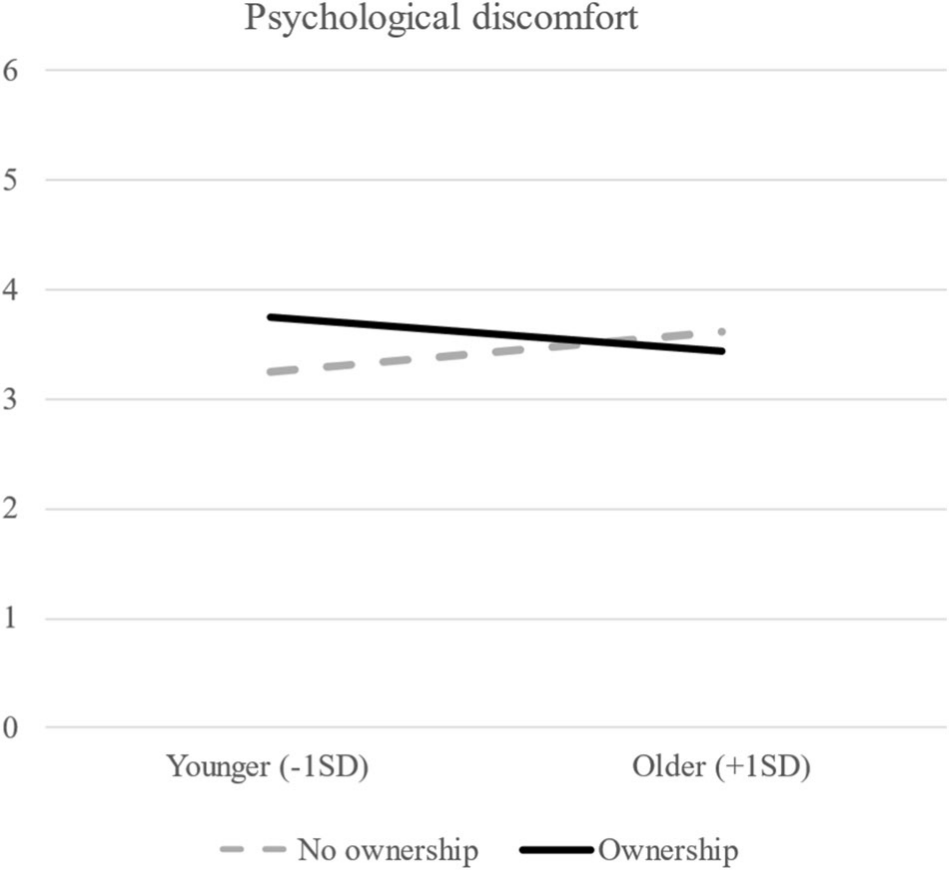
Psychological discomfort, as a function of age and ownership

The effect of device ownership (vs. no ownership) on psychological discomfort was statistically significant for younger minors (see Fig. 3) meaning that those younger participants who owned their electronic device (vs. no ownership) reported higher psychological discomfort; however, no differences were found for older participants. Lastly, the moderation analysis revealed a non-significant three-way interaction between age, active mediation, and ownership on dependent variables.

## Discussion

Despite considerable research on the detrimental effects of smartphone usage on the well-being of children and adolescents, the available evidence regarding smartphone ownership remains limited and often inconsistent. To date, there has been no research conducted to evaluate how smartphone ownership influences the well-being of minors through their consumption of content created by influencers. The present study begins to address this gap by examining the relationship between electronic device ownership, the consumption of influencer-generated content, and potential adverse outcomes such as psychological distress, problematic usage patterns, or emulation of risky behaviors among children and adolescents. Moreover, prior studies have assessed the effectiveness of preventive parental strategies in broader Internet usage contexts. However, how the ownership of an electronic device might impact their efficacy, particularly within the realm of influencer-generated content, remains unexplored. The present study contributes to this literature by examining whether different parental mediation strategies could mitigate potential adverse outcomes when device ownership is transferred to minors.

The findings revealed a positive association between smartphone ownership and some indicators of poorer well-being. In particular, the possession of an electronic device by minors is associated with the consumption of influencer-generated content, which in turn is positively correlated with the parasocial relationship minors develop with the influencer. As a result, minors experience increased psychological discomfort (i.e., feeling worse about their bodies, their lives, or about themselves), problematic usage (i.e., interfering with their friendships, sleeping time, or homework), and are more likely to imitate risky behaviors. Thus, in line with previous findings from similar research in the broader field of social network use in general (Hoffner & Bond, 2022), the data indicated that owning an electronic device, which provides direct and unlimited access to content, could be considered a facilitating factor in increased consumption of influencer-generated content and its consequent effects. Furthermore, these findings advance the understanding of how following and engaging with influencer-generated content might have a pervasive impact on minors at three levels: psychological (discomfort), developmental and social issues (problematic usage), and behavioral consequences (dangerous behaviors). Thus, a first recommendation derived from the present findings would be to invite parents and educators to promote a moderate consumption of social media in general and influencers in particular among children and adolescents. In addition, given the strong connection that followers establish with their favorite influencers and the relevance of that relationship in terms of influence, it is advisable for parents—as they do with friends in the offline world—to be equally vigilant about who these new virtual friends are and the messages, values, and behaviors they project.

Regarding age, the results showed that older children tend to display more problematic usage, probably because older age is also associated with a greater likelihood of owning an electronic device and with extended exposure to influencer-generated content. However, unlike other studies have suggested (Orben et al., 2022), the findings generally showed no moderation based on age, indicating a similar level of vulnerability across both children and adolescents. One possible explanation is that such age differences were identified using a longitudinal design (detecting differences one year after the initial measurement), whereas the current research is cross-sectional. An alternative explanation could be the difference in the content and behaviors analyzed. While previous research investigated general social media usage (encompassing various content types and purposes), this study specifically concentrates on the consumption of influencer-generated content. The only result moderated by age was that the detrimental effect of smartphone ownership on psychological discomfort was greater for younger children, suggesting that perhaps younger children are even more vulnerable when it comes to making social comparisons with influencers, as they—similar to the dynamics observed in influencer marketing as a whole (Loose et al., 2023)—may have fewer resources to understand and cope with the social image projected by their role models. Thus, the well-being indicators that might be affected by the consumption of influencer-generated content and smartphone ownership could vary depending on the child’s developmental stage. Therefore, considering these findings is crucial for enhancing interventions by parents and educators, as understanding the age dynamics can improve the effectiveness of efforts aimed at promoting the well-being and safety of children.

Regarding the effectiveness of the different parental mediation strategies examined, the data showed that the only strategy that showed a reverse relationship with the predicted negative outcomes was active mediation. This implies that discussing with minors the potential dangers and distortions of social media could prevent them from experiencing negative effects that impact their well-being. Specifically, it seems advisable to discuss how the content generated by influencers often portrays a physical and social image that does not reflect reality, as well as the risks associated with some of the behaviors they display in their posts. On the other hand, restrictive strategies regarding content (but not regarding time) showed a positive and significant correlation with each of the negative consequences reported by children. Thus, to the extent that children stated that their parents prohibited them from accessing specific influencers, they expressed greater psychological discomfort, problematic usage, and dangerous behavior imitation. Thus, content restrictive mediation may lead to unintended consequences, possibly because imposing restrictions could trigger active resistance and increase engagement with such content, as previous studies suggest (Meeus et al., 2018). These results suggest that attempting to implement any mediation strategy should be perceived by minors as part of a dialogue rather than as an imposition or prohibition, as this could elicit resistance or adverse reactions. Furthermore, this aligns with previous literature indicating that while an autonomy-supportive parenting style that respects adolescents’ feelings and preferences is associated with enhanced well-being, a controlling parenting style (i.e., authoritarian) has been linked to internalizing problems such as anxiety and depression, as well as externalizing problems such as behavioral issues in school and substance use (Young & Tully, 2022).

Importantly, the results indicated that the potential protective effect of active mediation was significantly weaker among children who owned an electronic device. Consequently, the effectiveness of this strategy could be reduced to the extent that the child has continuous and direct access to content. Therefore, the findings support the advice promoting thoughtful consideration when deciding whether to provide smartphones or similar electronic devices for the exclusive use of minors. In this regard, based on the existing empirical evidence, it is reasonable to suggest that parents’ choice to give or not give a smartphone or similar device to children could be considered as an additional form of parental mediation strategy.

It is important to mention that the correlational design of the study prevents definitive conclusions about the direction of these findings. For instance, the association found between the consumption of influencer-generated content and lower well-being indicators does not necessarily allow us to conclude that it is the longer amount of time of consumption that reduces well-being, but that an opposite alternative could also be possible. Future research employing experimental methodologies should establish causal relationships and clarify the direction of effects. In the same way, the positive association found between restrictive mediation and higher psychological discomfort, problematic usage, and imitation of dangerous behaviors could have an interpretation opposite to the one previously stated (i.e., restrictive mediation promotes an undesirable response leading to a more problematic usage of this type of content). Instead, the use of restrictive mediation could be a consequence of parental concern when perceiving that their child is experiencing such negative consequences. Therefore, longitudinal designs would be useful to elucidate the order in which these related variables influence each other. Another limitation to consider is that the present study relied on self-reports for all variables. Even though self-report measures are commonly used in similar research because of their reliability (Sandvik et al., 1993), they are sometimes susceptible to some biases (Rosenman et al., 2011). Thus, future research would benefit from the use —as far as possible— of a greater number of objective indicators. In addition, future studies could delve into the specific content and behaviors exhibited by highly followed influencers, allowing for a clearer understanding of whether these influencers engage in risky behaviors and the specific characteristics of such behaviors. Finally, this research has focused on the study of some of the possible negative consequences of influencer-generated content consumption, but it would be advisable that future research could also focus on the possible benefits that could be derived from the consumption of this type of content and, therefore, on the study of those mediation strategies that might reduce the risks without limiting such benefits.

## Conclusion

Children and adolescents are increasingly gaining possession of their own smartphones (or similar electronic devices) at younger ages. However, research on the potential effects that smartphone ownership could have on the well-being of minors, as well as its possible causes, remains quite limited. This study contributes to fill this gap by providing empirical support to the interplay between smartphone ownership, the consumption of influencer-generated content, the parasocial relationship with the influencer, and well-being. This study also investigated the effectiveness of commonly employed parental strategies and how they might differ in their impact when minors have their own smartphones. Results showed that the ownership of electronic devices was found to predict poorer indicators of well-being (i.e., psychological discomfort, problematic usage, and imitation of dangerous behaviors) among children and adolescents. Additionally, this association was explained by an increase in the consumption of influencer-generated content, leading to a subsequent stronger parasocial relationship with the influencer. This suggests that, regardless of age, the exposure to this kind of content—which parents often perceive as relatively harmless—could negatively impact the well-being of minors. In terms of preventive strategies, only active parental mediation showed an inverse relationship with negative well-being indicators. However, this beneficial effect diminished significantly when the minor owned an electronic device. These findings underscore the necessity for parents and educators to have open discussions with their children, elucidating the risks, threats, and falsehoods present in influencer-generated content. Additionally, the importance of carefully considering the decision to provide minors with a smartphone is emphasized.
